# Neural Crest Migration and Survival Are Susceptible to Morpholino-Induced Artifacts

**DOI:** 10.1371/journal.pone.0167278

**Published:** 2016-12-22

**Authors:** Elena F. Boer, Cicely A. Jette, Rodney A. Stewart

**Affiliations:** Department of Oncological Sciences, Huntsman Cancer Institute, University of Utah, Salt Lake City, Utah, United States of America; Texas A&M University, UNITED STATES

## Abstract

The neural crest (NC) is a stem cell-like embryonic population that is essential for generating and patterning the vertebrate body, including the craniofacial skeleton and peripheral nervous system. Defects in NC development underlie many birth defects and contribute to formation of some of the most malignant cancers in humans, such as melanoma and neuroblastoma. For these reasons, significant research efforts have been expended to identify genes that control NC development, as it is expected to lead to a deeper understanding of the genetic mechanisms controlling vertebrate development and identify new treatments for NC-derived diseases and cancers. However, a number of inconsistencies regarding gene function during NC development have emerged from comparative analyses of gene function between mammalian and non-mammalian systems (chick, frog, zebrafish). This poses a significant barrier to identification of single genes and/or redundant pathways to target in NC diseases. Here, we determine whether technical differences, namely morpholino-based approaches used in non-mammalian systems, could contribute to these discrepancies, by examining the extent to which NC phenotypes in *fascin1a (fscn1a)* morphant embryos are similar to or different from *fscn1a* null mutants in zebrafish. Analysis of *fscn1a* morphants showed that they mimicked early NC phenotypes observed in *fscn1a* null mutants; however, these embryos also displayed NC migration and derivative phenotypes not observed in null mutants, including accumulation of *p53*-independent cell death. These data demonstrate that morpholinos can cause seemingly specific NC migration and derivative phenotypes, and thus have likely contributed to the inconsistencies surrounding NC gene function between species. We suggest that comparison of genetic mutants between different species is the most rigorous method for identifying conserved genetic mechanisms controlling NC development and is critical to identify new treatments for NC diseases.

## Introduction

In vertebrate embryos, neural crest (NC) cells migrate along stereotypical pathways to form diverse derivatives, including elements of the craniofacial skeleton, cardiac tissue, pigment cells and neurons and glia of the peripheral nervous system [1]. Deficiencies in NC development, particularly cell migration and survival, underlies a large proportion of craniofacial abnormalities and congenital heart defects in children, called neurocristopathies, such as Waardenburg syndrome [2]. In addition, NC cells represent the origin of some of the most highly malignant cancers, including melanoma and neuroblastoma [3]. Thus, considerable efforts have been made to identify genetic pathways controlling NC development in order to discover disease-causing genes and identify new therapeutic targets.

Genetic studies in different model organisms such as mouse, zebrafish, chick and frog have identified genes that regulate one or more stages of NC development, such as induction, migration and differentiation [1]. Recent efforts have placed these genes into a conceptual framework, called the gene regulatory network (GRN), which organizes genes into pathways that control distinct phases of NC development [4, 5]. Thus, the GRN provides a list of gene products that can be potentially targeted for the treatment of NC diseases. However, comparative analysis of gene function between non-mammalian (fish, chick, frog) and mouse systems shows that genes within the NC GRN often do not have conserved functions in mammals. For example, the Snail family of transcription factors is required for NC epithelial-to-mesenchymal transition (EMT) in chick and frog embryos but is dispensable for NC EMT in mouse [6] (also see [7] for many more examples). The basis for this discrepancy is not known, but it has led to significant confusion surrounding the functional relevance of certain genes in the GRN to mammalian NC development and disease. While redundant genetic pathways have been proposed to account for these conflicting outcomes, another obvious difference between these model systems involves the method of genetic manipulation; most studies in non-mammalian systems have relied upon genetic knockdown approaches while knockouts have traditionally been used in mice. Here, we test whether technical differences could account for these discrepancies by determining the extent to which morpholino (MO)-based NC phenotypes mimic those exhibited by the corresponding null genetic mutant in zebrafish, currently the only system to directly compare such approaches.

MOs are a transient knockdown reagent that can be designed to recognize and bind the initiation codon or exon-intron junction of any mRNA transcript to interfere with translation (translation-blocking) or processing (splice-blocking), respectively [8]. Early reports demonstrated that MOs reduced gene function in an efficient and specific manner; several MOs that were tested produced developmental defects that faithfully recapitulated the phenotypes observed in the corresponding genetic mutant [9]. Since then, MOs have became a common tool to rapidly analyze gene function in zebrafish and have been used to study an array of developmental processes, including gastrulation, morphogenesis and organ development [8, 10]. However, the propensity for MOs to induce off-target phenotypes, such as nonspecific activation of the p53 signaling pathway [11], has also become increasingly clear. In an effort to circumvent these limitations, a set of guidelines for the use of MOs was established such that in cases where these strict criteria could be met, MOs would still represent a powerful tool for rapid analysis of “double mutant” or haploinsufficient phenotypes [8, 12]. However, as stable targeted-mutagenesis approaches (via TALENs or CRISPR/Cas9) have recently become widely available in zebrafish, many genetic mutants have been created [13–15]. Consequently, comparisons between genetic mutant and morphant phenotypes have revealed that morphant phenotypes include false-positive and false-negative features not observed in the corresponding genetic mutant [16–19]. These analyses suggest MOs should only be utilized in cases where they have been shown to precisely mimic the selective phenotypes observed in the corresponding genetic mutants [15, 16].

Previously, we demonstrated that maternal/zygotic null mutations in zebrafish *fascin1a (fscn1a MZ)*, a gene that encodes a filopodia protein called Fascin1, result in partially penetrant defects in the directional migration of a subset of NC cells [20]. Concomitant with abnormal directional NC migration, ~20% of *fscn1a MZ* embryos display defects in the first (mandibular) arch, but not posterior arches, of the craniofacial skeleton as well as a reduction in NC-derived sympathetic and enteric neurons, but not dorsal root ganglia neurons. Thus, the selective defects in NC migration and derivative formation observed in the *fscn1a* MZ mutant line provide rigorous criteria by which to define whether a MO can truly recapitulate the defects of a NC mutant.

In this study we analyzed a translation-blocking MO designed to target *fscn1a* (*fscn1aMO*). The *fscn1aMO* efficiently reduced Fscn1a protein levels, and *fscn1a* morphant phenotypes were rescued by exogenous non-MO-targeted wild-type *fscn1a* mRNA. In addition, *fscn1a* morphants precisely phenocopied the defective filopodia feature of migrating NC cells that are observed in *fscn1a MZ* mutant embryos. However, at later stages, the *fscn1aMO* caused defects in NC derivatives that were either more severe than, or not observed in, *fscn1a MZ* animals, such as loss of posterior craniofacial elements and dorsal root ganglia neurons. We also found that the *fscn1aMO* induced *p53-*independent cell death throughout the head and NC. Our results demonstrate that even under “ideal” experimental conditions, a MO can cause NC phenotypes that are not observed in null mutants, with collective NC migration behaviors and survival being exceptionally sensitive. Thus, MO-induced artifacts have likely contributed to the inconsistent findings concerning the genetic regulation of NC development between species, particularly in cases in which NC phenotypes are observed in non-mammalian systems but not in mice. We suggest that comparative genetic mutant analyses are required to clarify the mechanisms controlling NC development *in vivo*, including the unambiguous identification of redundant pathways, to ultimately guide rationally-designed therapies for NC diseases in humans.

## Materials and Methods

### Ethics statement

All experiments involving zebrafish were approved by and conformed to the regulatory standards and guidelines of the University of Utah Institutional Animal Care and Use Committee (IACUC#16–03019). Zebrafish embryos were between 24 hours post fertilization (hpf) and 120 hpf depending on the experiment, with the exact age indicated in each of Figure. Zebrafish adults (between 1–2 years of age) were used only for breeding purposes. In accordance with the IACUC standards, zebrafish embryos or adults were euthanized by immobilization by submersion in ice water (5 parts ice/1 part water) for at least 10 minutes following cessation of opercular movement and then placed in a freezer, as approved by IACUC protocol #16–03019. The oldest age at which the zebrafish were sacrificed in this study is 120 hpf (embryos) or 2 years old (adults).

### Zebrafish husbandry and transgenic animals

Zebrafish were maintained and bred as described [21]. The *fscn1a MZ* mutants were previously described [20]. Transgenic lines used were described previously: *Tg(sox10*:*rfpmb)* [22], *Tg(sox10*:*gfp)* [23], *Tg(ngn1*:*gfp)* [24], *Tg(phox2b*:*gfp)* [25].

### Molecular biology and cloning

To generate the *fscnMOgfp* reporter construct, the GFP coding sequence was PCR amplified and cloned into pCS2; the GFP forward primer included an overhang that consisted of the 25 nucleotide sense *fscn1aMO* target sequence. pCS2-*fscn1a* was described previously [20, 26]. To generate a non-targeted *fscn1a* mRNA for rescue experiments with the *fscn1a-ATGMO*, wild-type zebrafish *fscn1a* was amplified with SuperScript one-step RT-PCR (Invitrogen) using a forward primer containing 7 silent base pair mismatches: *fscn1a-F(mut)*- ATGACcGCtAAtGGtACtAGtGAtA (silent wobble mismatches are denoted in lower case). PCR products were cloned into pGEM-T Easy (Promega) and sequenced. Verified *fscn1a* clones containing 7 silent mutations were then directionally subcloned into pCS2 for generating mRNA.

### Immunoblotting

Embryonic protein lysates were prepared as previously described [20]. Antibodies used in this study were rabbit anti-FSCN1 (1:2000, Sigma) and mouse anti-GAPDH (1:1000, Abcam).

### Alcian blue staining, whole-mount in situ hybridization, acridine orange staining and activated Caspase-3 immunofluorescence

Cartilage staining with Alcian blue and whole-mount *in situ* hybridization was carried out as previously described [27, 28]. Antisense RNA probes were generated for *foxd3*, *crestin*, *dlx2a*, *sox10*, and *th* as described [29]. Acridine orange staining and whole-mount immunofluorescence for activated Caspase-3 or GFP was performed as previously described [20, 30, 31].

### Morpholino and mRNA microinjection

Based on the published GenBank sequence of *fscn1a* (Gene ID: 558271), custom translation-blocking (*fscn1a-ATGMO*, tgtcgctggttccgtttgcagtcat) and splice-blocking (*fscn1a-i1e2MO*, catgcctgacaaacacaagatcgac) MOs were designed and synthesized by GeneTools, LLC. The *tp53MO* is described elsewhere [11]. A non-targeting control MO (*coMO*) was ordered from GeneTools, LLC. To generate full-length capped sense mRNA, pCS2-*fscn1a* or pCS2-*fscnMOgfp* was 1) linearized with Not1 and 2) transcribed *in vitro* with SP6 mMESSAGE mMACHINE kit (Ambion). Morpholinos or mRNA were microinjected into the yolk of one-cell embryos at the following dosages: 0.5ng, 0.625ng or 1.25ng *fscn1a-ATGMO*, 0.625ng or 1.25ng *fscn1a-i1e2MO*, 0.75ng *tp53MO*, 50pg *fscnMOgfp* mRNA, 25pg *fscn1a* mRNA. For rescue experiments, full-length *fscn1a* mRNA that does not contain the MO-binding site was premixed with MO and loaded into the same needle before injections.

### Image acquisition and processing

Confocal images were acquired using an Olympus Fluoview FV1000XY, FV10i or FV1200 confocal microscopes and Olympus FV10-ASW v4.1 software. All imaging was performed using Olympus UPlanSApo 60X water and Olympus UPlanSApo 10X objectives. Embryos were embedded in 1% low melt agarose on cover slips for all confocal imaging. For analysis of filopodia dynamics, z-stacks of the leading edge of NC stream 3 in 26 hpf *Tg(sox10*:*rfpmb)* embryos injected with *tp53MO* or *tp53MO* plus *fscn1aMO* (n = 5 of each) were acquired every 2 minutes for 1 hour using the 60X water objective. For analysis of NC stream depth, z-stacks were acquired of NC stream 3 in 26 hpf *Tg(sox10*:*rfpmb; sox10*:*h2a-gfp)* embryos injected with *tp53MO or tp53MO* plus *fscn1aMO*. Cranial NC migration was imaged in 22, 25, 28 and 36 hpf *Tg(sox10*:*GFP)* embryos using a Zeiss Axiovert 200 inverted microscope configured with an Olympus DP72 camera. Widefield fluorescent images were acquired on an Olympus SZX16 microscope configured with an Olympus DP72 camera. Brightfield images were taken using a Nikon C-DSD115 microscope configured with an Olympus DP72 camera. Prism 6, ImageJ 1.46r, Adobe Photoshop CC 2014–2015, and Adobe Illustrator CC 2014–2015 were used to generate figures.

### Quantitative real-time PCR

Standard methods for RNA isolation, cDNA synthesis and quantitative PCR were used and are described elsewhere [26]. Primers were designed by Roche to be used with the Universal Probe Library; primer sequences and probes are listed in S1 Table. Data are combined from three independent experiments and were plotted in Prism 6. Statistical analysis was performed using an unpaired t-test (Prism 6). Error bars represent SEM. Significance is denoted with asterisks: *p = 0.0057.

### Quantification and statistical analysis

To quantify flopodia in *tp53*^*zdf1*^ and *fscn1a* morphant embryos, filopodia at the leading edge of cranial NC stream 3 were analyzed using ImageJ 1.46r. All filopodia within a 20 μm region were counted and measured. For each experimental condition, 5 filopodial protrusions from 5 embryos were analyzed (n = 25). *tp53*^*zdf1*^ and *fscn1a* morphant filopodia were compared using an unpaired t-test (Prism 6). Significance is denoted with asterisks: **p<0.005, ***p<0.001.

## Results

### The specificity of *fscn1a*-targeted antisense morpholinos was validated by standard tests

In zebrafish, *fscn1a* zygotic (*fscn1a zyg*) mutants are viable and fertile with no obvious phenotypes due to perdurance of maternally-derived Fscn1a mRNA and protein throughout embryonic development [20]. In contrast, ~20% of embryos lacking maternal and zygotic *fscn1a* (*fscn1a MZ)* display defects in neural crest (NC) cell migration and subsequent derivative formation. Because analyzing *fscn1a MZ* phenotypes for genetic interactions requires at least three generations of genetic crosses (~12 to 18 months), we sought to determine if antisense morpholino (MO) oligonucleotides could be used in zebrafish embryos to inhibit maternal and zygotic *fscn1a* function and thereby recapitulate the NC defects observed in *fscn1a MZ* mutants more rapidly.

**Fig 1:**
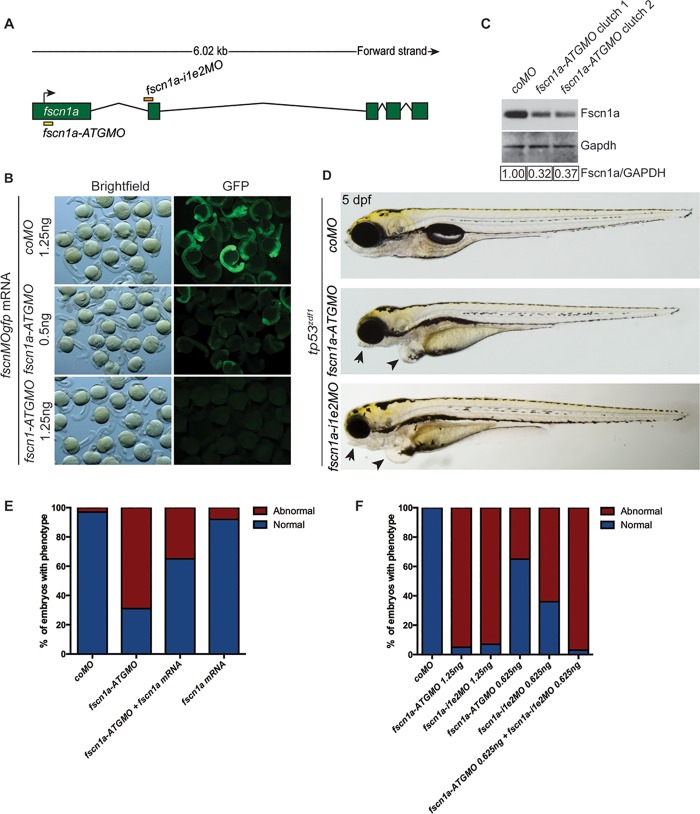
Analysis of a translation-blocking *fscn1a* morpholino. (A) Schematic of *fscn1a* genomic locus. Binding sites for translation-blocking *fscn1a-ATGMO* and splice-blocking *fscn1a-i1e2MO* are depicted. Arrow indicates translation start site. (B) Bright-field and fluorescent images of 24 hpf *tp53*^*zdf1*^ mutant embryos injected with the indicated amount of *fscnMOgfp* mRNA and *coMO* or *fscn1a-ATGMO*. (C) Immunoblot showing Fscn1a protein levels in 48 hpf *tp53*^*zdf1*^ embryos injected with 1.25 ng of *coMO* or *fscn1a-ATGMO*. Values below the blot represent relative band intensity of Fscn1a/GAPDH normalized to *coMO* sample. (D) Representative bright-field images of 5 dpf *tp53*^*zdf1*^ mutant embryos injected with 1.25 ng of the indicated MOs. Fscn1a morphants show loss of tissue associated with the lower jaw as well as cardiac edema (arrows highlight both phenotypes). (E) *tp53*^*zdf1*^ embryos were injected with the indicated MO and analyzed at 5 dpf for craniofacial morphology. (F) *tp53*^*zdf1*^ embryos were injected with the indicated MO (1.25 ng) and/or mRNA (25 pg) and analyzed at 5 dpf for craniofacial morphology. All experiments in this figure were performed independently at least three times with similar results.

Based on the published GenBank sequence for *fscn1a*, a MO was designed that is complementary to the translation start site of *fscn1a* (translation-blocking, *fscn1a-ATGMO*). The *fscn1a-ATGMO* is expected to bind and inhibit the translation of both maternal and zygotic *fscn1a* mRNA transcripts (Fig 1A). The optimal dose of *fscn1a-ATGMO* was determined by a standard phenotype-based dose response. The efficiency of *fscn1a-ATGMO* binding to its target sequence was evaluated by determining the ability of *fscn1a-ATGMO* to reduce fluorescence of an mRNA construct composed of the 25-nucleotide *fscn1a-ATGMO* target sequence fused to the GFP coding sequence (*fscnMOgfp*). Co-injection of *fscn1a-ATGMO* and *fscnMOgfp* into one-cell stage zebrafish embryos resulted in a reduction (0.5ng) or complete loss (1.25ng) of GFP fluorescence relative to expression of *fscnMOgfp* alone (Fig 1B), indicating that the *fscn1a-ATGMO* can effectively target the complementary mRNA sequence to inhibit *gfp* mRNA translation.

To determine if *fscn1a-ATGMO* could inhibit *fscn1a* translation, we evaluated Fscn1a protein levels in 48 hpf embryos. Compared to embryos injected with a non-targeting control MO (*coMO*), injection of 1.25 ng of *fscn1a-ATGMO* efficiently reduced Fscn1a protein levels (Fig 1C). Higher doses of the *fscn1a-ATGMO* did not reduce Fscn1a protein levels further. As we previously showed that unfertilized zebrafish oocytes express both *fscn1a* mRNA and Fscn1a protein [20], we concluded that the residual Fscn1a protein (Fig 1C) is maternally-derived and cannot be targeted by MOs.

Approximately 20% of *fscn1a MZ* mutant embryos display a specific loss of NC-derived craniofacial elements formed from the first (mandibular) arch [20]. To determine whether the *fscn1a-ATGMO* could phenocopy *fscn1a MZ* mutants, we injected 1.25 ng of *fscn1a-ATGMO* into wild-type or *tp53*^*zdf1*^ embryos (to control for potential MO-induced nonspecific p53-dependent apoptosis [11]) at the one-cell stage of development. At 5 days post-fertilization (dpf), ~65 to 100% of *tp53*^*zdf1*^ embryos injected with *fscn1a-ATGMO* displayed morphological abnormalities that are commonly used as evidence of defective NC development, including loss of tissue associated with the lower jaw, lack of jaw extension and mild cardiac edema (Fig 1D, arrows and arrowheads, respectively). Thus, the *fscn1a-ATGMO* induced more severe and more penetrant craniofacial abnormalities than those observed in the *fscn1a MZ* mutants, as well as heart defects that were not observed in the mutants.

Historically, to test for specificity of MO-induced phenotypes in the absence of a corresponding genetic mutant, a second MO is employed to corroborate the phenotypes observed in response to the first MO. We therefore designed a second MO to target the intron 1 –exon 2 boundary in *fscn1a* pre-mRNA and block normal splicing (*fscn1a-i1e2MO*, Fig 1A). Because splice-blocking MOs function by preventing mRNA splicing, they are effective only against zygotic mRNA transcripts [9]. The *fscn1a zyg* genetic mutants are phenotypically normal, so it was surprising that injection of 1.25 ng of *fscn1a-i1e2MO*, which only targets zygotic transcript, resulted in morphological abnormalities similar to those observed in embryos injected with the translation-blocking MO (Fig 1D and 1E). At a reduced dose (0.625 ng), injection of either *fscn1a-ATGMO* or *fscn1a-i1e2MO* caused morphological defects at a lower penetrance (Fig 1E). However, simultaneous injection of both MOs at the lower dose elicited an additive effect and increased the penetrance of MO-induced phenotypes to that observed in embryos injected with a higher dose of either *fscn1a*-targeted MO (Fig 1E). Thus, without knowledge of the *fscn1a MZ* mutant phenotype, it would have appeared as though each MO was specifically inhibiting expression from the *fscn1a* gene and that *fscn1a* was required for overall NC development.

The next standard test for specificity was to determine whether the MO-induced phenotypes could be rescued with mRNA in which the MO target site has been mutated (albeit silently in regard to protein coding) to prevent binding to the MO. We co-injected non-MO-targeted *fscn1a* mRNA with the *fscn1a-ATGMO* and found that *fscn1a* mRNA rescued morphological defects in a subset of 5 dpf *fscn1a* morphant embryos (Fig 1E). By every measure, in the absence of comparison to a genetic mutant, the MO-induced morphological NC defects appeared to be specific to knockdown of the *fscn1a* gene.

### The *fscn1aMO* phenocopied the defective filopodia formation in NC cells observed in the *fscn1a MZ* mutant

**Fig 2:**
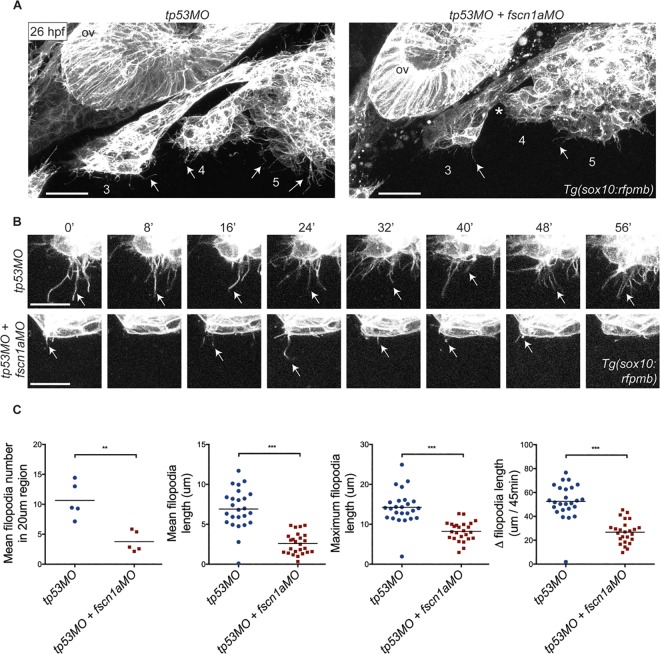
*fscn1aMO* reduces NC-cell filopodia formation. (A) Lateral views of posterior cranial NC streams in 26 hpf *Tg(sox10*:*rfpmb)* embryos injected with *tp53MO* or *tp53MO* plus *fscn1aMO*. Numbers correspond to NC streams. Arrows denote filopodia at leading edge of NC streams. Arrowheads mark RFP-positive puncta surrounding NC streams. Asterisk highlights fusion of NC streams 3 and 4 in *fscn1a*-morphant embryo. ov; otic vesicle. Scale bar = 50μm. (B) Time-lapse confocal images of filopodia at leading edge of NC stream 3 in 26 hpf *Tg(sox10*:*rfpmb)* embryos injected with *tp53MO* or *tp53MO* plus *fscn1aMO*. Arrows mark tips of single filopodia throughout the time lapse. Scale bar = 10 μm. (C) Quantitation of mean filopodia number in 20 μm region at leading edge of NC stream 3, mean filopodia length, maximum filopodia length, and change in filopodia length over 45 minutes in 26 hpf *Tg(sox10*:*rfpmb)* embryos injected with *tp53MO* or *tp53MO* plus *fscn1aMO* (n = 5 embryos and 25 filopodia for each condition, **p<0.005, ***p<0.001 by an unpaired t-test analysis). In all panels, anterior is to the left. At least three independent experiments were performed in this figure with similar results.

We next analyzed the extent to which the *fscn1a-ATGMO* (hereafter referred to as *fscn1aMO*) could induce NC defects that were specifically due to loss of *fscn1a*. Filopodia formation is severely impaired in *fscn1a MZ* NC cells [20]. To determine if the *fscn1aMO* also disrupts NC-cell filopodia formation, we injected *tp53MO* or co-injected *tp53MO* and *fscn1aMO* into *Tg(sox10*:*rfpmb)* embryos in which *sox10*-expressing NC cells are labeled with a membrane-bound RFP to visualize NC cellular protrusions [22]. At 28 hpf, *Tg(sox10*:*rfpmb)* embryos injected with *tp53MO* displayed numerous filopodial protrusions at the leading edge of all cranial NC streams (Fig 2A–2C). In *tp53MO* embryos, NC-cell filopodia were highly dynamic and displayed significant extension and retraction over the course of 60 minutes (Fig 2B and 2C). In contrast, filopodia at the leading edge of cranial NC streams in *Tg(sox10*:*rfpmb)* embryos injected with *fscn1aMO* were significantly reduced in number, length and dynamics (Fig 2A–2C, arrows). Throughout these live imaging studies, we noted the appearance of RFP-positive puncta (arrowheads) within and surrounding *fscn1a* morphant cranial NC streams (Fig 2A), which was suggestive of dying cells. Thus, loss of *tp53* did not appear to ameliorate the phenotypes caused by *fscn1aMO*, including cell death. In addition, contact between posterior *fscn1a* morphant cranial NC streams was frequently observed (Fig 2A, asterisk). Together, these phenotypes are indicative of a defect in cell survival and migration of cranial NC streams in *fscn1a* morphant embryos. With respect to filopodia defects, the *fscn1aMO* closely phenocopied the *fscn1a* MZ mutant line. Numerous studies demonstrating that Fscn1 is required for filopodia formation *in vitro* [32] support the interpretation that the *fscn1aMO* is functioning specifically and selectively to control NC cell behaviors during development.

### *Fscn1a* morphant embryos displayed defects in NC migration

**Fig 3:**
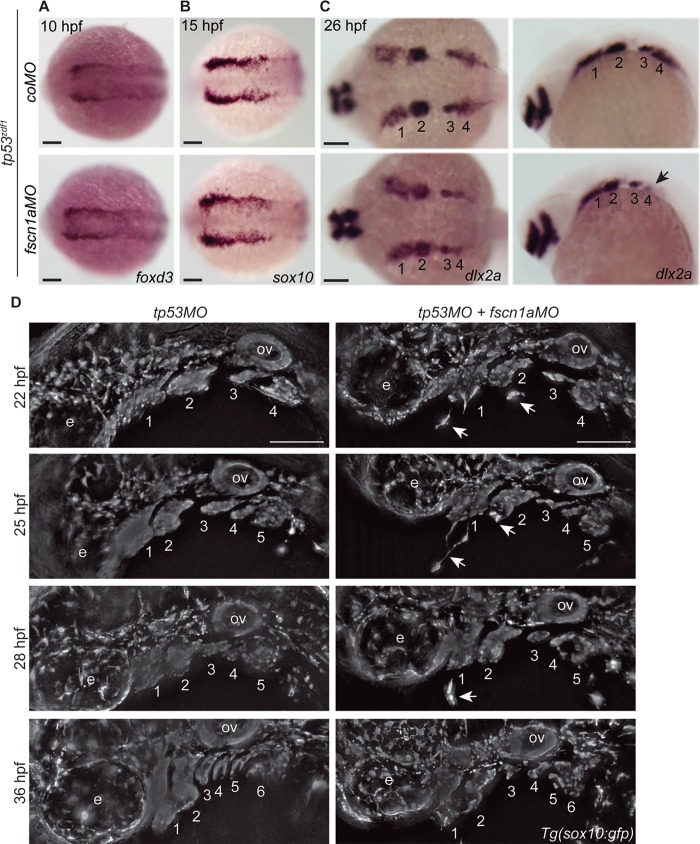
Late-stage NC-cell migration is disrupted in *fscn1a*-morphant embryos. (A-B) Dorsal cranial views of *tp53*^*zdf1*^ embryos injected with *coMO* or *fscn1aMO* and analyzed by whole-mount *in situ* hybridization (ISH) for (A) *foxd3* mRNA at 10 hpf and (B) *sox10* mRNA at 15 hpf. (C) Dorsal cranial and lateral views of 26 hpf *tp53*^*zdf1*^ embryos injected with *coMO* or *fscn1aMO* and analyzed by whole-mount ISH for *dlx2a*. Numbers correspond to pharyngeal arches. Arrow denotes reduction in *dlx2a*-positive cranial NC cells in *fscn1a* morphants. (D) Lateral views of cranial NC streams in 22, 25, 28 and 36 hpf *Tg(sox10*:*gfp)* embryos injected with *tp53MO* or *tp53MO* plus *fscn1aMO*. Numbers correspond to NC streams. Arrows highlight NC cells migrating independently of NC streams in *fscn1a* morphants. e; eye, ov; otic vesicle. In all panels, anterior is to the left. All experiments in this figure were performed independently at least three times with similar results. All scale bars in this figure = 100 μm.

We recently demonstrated that *fscn1a MZ* genetic mutant embryos display partially penetrant defects in directional NC-cell migration. In ~20% of *fscn1a MZ* embryos, the first cranial NC stream migrated outside of normal stream boundaries and an overall reduction in *dlx2a*-positive NC cells was observed [20]. To determine if defective filopodia formation resulted in abnormal NC development in *fscn1a* morphant embryos, we first analyzed the spatial expression pattern of *foxd3*, a transcription factor required for NC specification, which serves as an early marker of premigratory NC cells [29]. In 11 hpf *tp53*^*zdf1*^ mutant embryos, *foxd3* expression was detected by whole-mount in situ hybridization (ISH) and was indistinguishable between *coMO* and *fscn1aMO*-injected embryos (Fig 3A), demonstrating that the *fscn1aMO* does not affect NC induction and/or specification. At 15 hpf, a time point that marks the earliest stages of cranial NC migration, expression of *sox10* in *tp53*^*zdf1*^ mutant embryos was evaluated by ISH; no differences in *sox10* expression were observed between *coMO* and *fscn1aMO-*injected embryos (Fig 3B), suggesting that early NC migration is normal in embryos injected with the *fscn1aMO*, consistent with *fscn1a MZ* genetic mutants.

To determine if the *fscn1aMO* disrupts later stages of NC migration, we analyzed the spatial expression pattern of *dlx2a*, a marker of migratory cranial NC cells destined for the pharyngeal arches, in *tp53*^*zdf1*^ mutant embryos injected with either *coMO* or *fscn1aMO* by ISH. In 26 hpf *fscn1a* morphant embryos, a strong reduction in *dlx2a*-expressing cranial NC cells in stream 4, which ultimately gives rise to posterior cartilage elements, was observed (Fig 3C). These defects could result from deficiencies in NC migration and/or abnormal patterning of the endoderm, mesoderm or ectoderm that forms the pharyngeal pouches [33]. Although both *fscn1a* morphant and *fscn1a MZ* embryos display abnormal *dlx2a* patterning, the defects in *fscn1a MZ* mutants were specific to the first NC stream (compare to Fig 4 in [20]). Therefore, the first clear evidence of inconsistency between *fscn1aMO-*induced and mutant-generated NC phenotypes was observed during analysis of collective NC-cell migration behaviors.

To conduct a detailed analysis of cranial NC migration in *fscn1a* morphant embryos, we injected *tp53MO* (control) or *tp53MO* plus *fscn1aMO* into *Tg(sox10*:*gfp)* embryos [23] and imaged cranial NC streams throughout NC migration. From 22- to 36 hpf, cranial NC cells in *Tg(sox10*:*gfp); tp53* morphant embryos migrate in distinct and stereotypical collective streams (Fig 3D). All streams migrate ventrally to invade the pharyngeal pouches by 36 hpf. At all time points evaluated, *fscn1a* morphant embryos displayed several defects in cranial NC-cell migration. In general, *fscn1a* morphant cranial NC streams appeared to be populated by fewer *sox10*-expressing NC cells and exhibited a disorganized morphology. In 22, 25 and 28 hpf *fscn1a* morphant embryos, single cranial NC cells were observed migrating outside of normal NC stream boundaries without contact with nearby NC streams (Fig 3C). At 36 hpf, *fscn1a* morphant NC streams appeared disordered and severely reduced in size, and failed to migrate ventrally as compared to *tp53* morphant NC streams (Fig 3C). Overall, the phenotypes observed in *fscn1a* morphant embryos demonstrate that the *fscn1aMO* causes a defect in directional and collective cranial NC migration. However, these seemingly-specific NC phenotypes are not consistent with the *fscn1a MZ* genetic mutant, as the primary defects in the genetic mutant are restricted to the first NC stream at the time points analyzed [20].

### Neural crest derivatives were defective in *fscn1a* morphant embryos

In the developing embryo, NC cells migrate extensively to form diverse derivatives, including elements of the craniofacial skeleton, neurons and glia of the peripheral nervous system and pigment-producing cells [1]. Approximately 20% of *fscn1a MZ* embryos display specific defects in a subset of NC-derived tissues, including a loss of cartilage elements formed from the most anterior (mandibular) pharyngeal arch, and a subtle, but statistically significant, decrease in *tyrosine hydroxylase* (*th*) and *dopamine β-hydroxylase* (*dbh*)-expressing peripheral neurons and *paired-like homeobox 2b* (*phox2b*)-expressing enteric neurons [20]. The formation of trunk NC-derived dorsal root ganglia (DRG) was unaffected in *fscn1a MZ* genetic mutants [20]. To determine if *fscn1a* morphant embryos display similar defects in NC derivatives, we analyzed the formation of several NC derivatives, including the craniofacial skeleton, *neurogenin1* (*ngn1*)-expressing DRG, *th*-positive sympathetic ganglia, *phox2b*-positive enteric neurons and pigment-producing melanophores.

**Fig 4:**
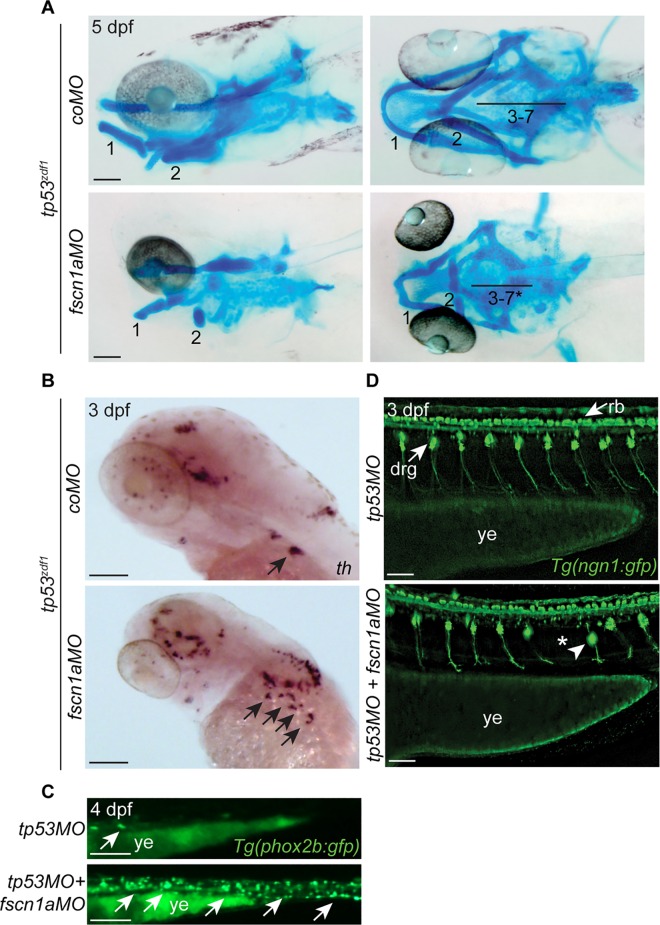
NC-derived tissues form abnormally in *fscn1a*-morphant embryos. (A) Lateral and ventral views of 5 dpf *tp53*^*zdf1*^ embryos injected with *coMO* or *fscn1aMO* and stained with Alcian blue. Numbers correspond to pharyngeal arches. Asterisk denotes arches that are severely reduced in size or absent. (B) Lateral views of 3 dpf *tp53*^*zdf1*^ embryos analyzed by whole-mount ISH for *th*. Arrows denote *th*-positive neurons of sympathetic ganglia. (C) Lateral views of section of the gut in 4 dpf *Tg(phox2b*:*gfp)* embryos injected with *tp53MO* or *tp53MO* plus *fscn1aMO*. Arrows denote *phox2b*-positive enteric neurons. (D) Lateral views of trunk in 3 dpf Tg(*ngn1*:*gfp*) embryos injected with *tp53MO* or *tp53MO* plus *fscn1aMO*. Arrows in top panel highlight *ngn1*-positive NC-derived dorsal root ganglia (drg) and central nervous system (CNS)-derived Rohon-Beard neurons (rb). In lower panel, arrowhead and asterisk indicate misplaced and absent dorsal root ganglia, respectively. drg; dorsal root ganglia, rb; Rohan-Beard neurons, ye; yolk extension. In all panels, anterior is to the left. All experiments in this figure were performed independently at least three times with similar results. All scale bars in this figure = 100 μm.

At 5 dpf, *fscn1a*-morphant embryos displayed several morphological abnormalities, including overt defects in craniofacial skeleton formation and cardiac edema (Fig 1D). At this stage, patterning of NC-derived melanophores appeared normal (Fig 1D). To further characterize the craniofacial skeleton, *tp53*^*zdf1*^ embryos were injected with *coMO* or *fscn1aMO*, fixed at 5 dpf, and stained with Alcian blue. In *fscn1a* morphant embryos, Alcian blue staining revealed defects in craniofacial structure, including ventral displacement of Meckel’s cartilage (mandibular arch), malformation of pharyngeal arches, and a substantial reduction in size of all arches (Fig 4A). The defects in craniofacial morphogenesis in *fscn1aMO* embryos are consistent with the aberrant cranial NC migration defects observed earlier in development, particularly the severe reduction in posterior NC streams, which is not observed in *fscn1a MZ* genetic mutants. Notably, the selective loss of cartilage elements associated with the mandibular jaw observed in *fscn1a* MZ mutants was likely obscured in the *fscn1a* morphants by *fscn1aMO*-induced widespread patterning defects in the head. This morphant phenotype would have therefore impeded the discovery that Fscn1a specifically controls directional collective migration of the first NC stream and that *fscn1a* MZ mutants represent an animal model for hemifacial microsomia.

We next analyzed the formation of NC-derived peripheral neurons in *fscn1a* morphant embryos. To determine if the development of peripheral sympathetic ganglion neurons were disrupted by the *fscn1aMO*, we performed ISH for *th* in 3 dpf *tp53*^*zdf1*^ mutant embryos injected with *coMO* or *fscn1aMO*. In *fscn1a* morphants, *th*-positive sympathetic ganglion neurons were disorganized and failed to aggregate into discrete ganglia (Fig 4B). Although *fscn1a* morphants showed a reduction in *th*-positive sympathetic ganglia similar to *fscn1a MZ* genetic mutants [20], the phenotype was more severe and occurred at a higher penetrance in *fscn1a* morphants. To analyze the formation of vagal NC-derived *phox2b*-positive enteric neurons, we injected *tp53MO* (control) or *tp53MO* plus *fscn1aMO* into *Tg(phox2b*:*gfp)* embryos at the one-cell stage [25]. At 3 dpf, *phox2b*-positive enteric neurons were almost completely absent in the developing gut of *fscn1a* morphant embryos (Fig 4C). Because NC stream 4 contributes significantly to the vagal NC, this dramatic loss of enteric neurons was consistent with the severe reduction in NC stream 4 *dlx2a*-positive cells observed in *fscn1a* morphants (Fig 3C). Thus, with respect to peripheral sympathetic and enteric neurons, the *fscn1aMO* caused more severe and penetrant defects compared to *fscn1a MZ* mutant embryos, which have defects in these neurons in only ~20% of embryos.

In the trunk, bilateral sensory DRG are formed from collective NC chains that migrate ventrally through each somite [1]. To determine if the *fscn1aMO* affects DRG formation, *tp53MO* or *tp53MO* plus *fscn1aMO* was injected into *Tg(ngn1*:*gfp)* embryos at the one-cell stage [24]. Compared to *tp53* morphant embryos, *fscn1a*-morphant DRG patterning was disorganized at 3 dpf. In some instances, the DRG were positioned abnormally with respect to the D/V axis (Fig 4D, arrowhead). In addition, single DRG were absent in many *fscn1a* morphant embryos (Fig 4D, asterisk). In contrast to *fscn1a* morphant embryos, no defects in DRG formation were observed in *fscn1a MZ* mutant embryos [20]. These data show that the *fscn1aMO* caused severe and distinct peripheral nervous system phenotypes that are absent in the null genetic mutant.

### *Fscn1aMO* promoted *tp53*-independent cell death

To account for the frequent observation that morphants show more severe phenotypes than the associated mutant, Rossi et al. [17] proposed that genetic compensation through activation of redundant gene functions in the mutant could mask phenotypes that are functionally relevant to the gene of interest. If this is the case, then the mutant embryo will confer a dominant masking effect in the presence of the morpholino, thus providing a straightforward way to test for this phenomenon. To this end, we injected the *fscn1aMO* into *fscn1a MZ* mutant embryos and found that the mutant was unable to rescue *fscn1aMO*-induced phenotypes (S1 Fig). This indicates that redundant genetic pathways are not upregulated in *fscn1a MZ* embryos and that the more severe phenotypes observed in the *fscn1a* morphants are likely due to off-target effects.

**Fig 5:**
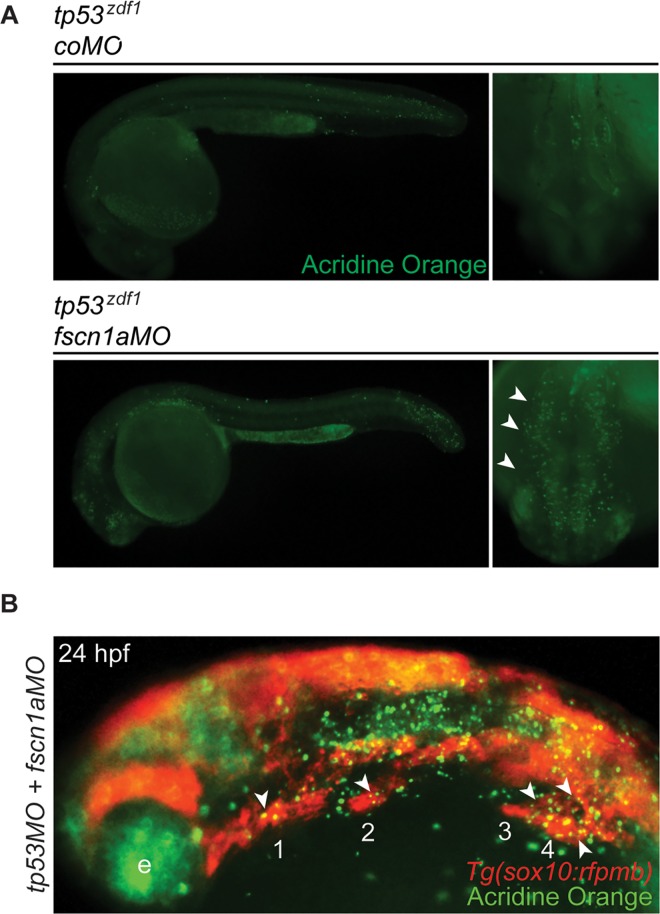
*Fscn1aMO* induced *tp53*-independent cell death in NC cells. **(A)** Lateral and dorsal cranial views of 28 hpf *tp53*^*zdf1*^ mutant embryos injected with *coMO* or *fscn1aMO* and stained with AO. Arrowheads highlight AO-positive cells adjacent to neural tube. **(B)** Lateral view of 24 hpf *Tg(sox10*:*rfpmb)* embryo injected with *tp53MO* plus *fscn1aMO* and stained with AO. Numbers correspond to NC streams. Arrows indicate regions of RFP-positive/AO-positive cells. e; eye, nt; neural tube. In all lateral views or dorsal cranial views, anterior is to the left or bottom, respectively. Experiments in this figure were performed independently at least three times with similar results.

To determine the mechanism causing the *fscn1aMO*-induced off-target effects, we analyzed cell survival, as indications of cell death were observed in *fscn1a* morphant embryos in live imaging experiments (Fig 2A, arrowheads) but none were detected in *fscn1a MZ* embryos (S2 Fig). Although the experiments described above were performed in a *tp53*-deficient background to circumvent MO-induced p53-mediated apoptosis [11], it was possible that the *fscn1aMO* elicited off-target cell death through alternative p53-independent signaling pathways to cause the severe and highly penetrant NC phenotypes observed in *fscn1a* morphants. To test this, we injected *coMO* or *fscn1aMO* into *tp53*^*zdf1*^ mutant embryos and stained 28 hpf embryos with acridine orange (AO), which detects cell death occurring by various mechanisms in live embryos [30] (Fig 5A). Compared to control embryos, *fscn1a* morphants displayed excessive AO-expressing cells, particularly in the cranial region (Fig 5A, arrowheads). To determine if the AO-positive cells were NC cells, we injected *tp53MO* plus *fscn1aMO* into *Tg(sox10*:*rfpmb)* embryos and stained with AO at 28 hpf. Although AO-positive cells were widespread in *fscn1a*-morphant embryos, a subset of AO-positive cells were also RFP-positive (Fig 5B), demonstrating that the *fscn1aMO* promotes cell death in a subset of NC cells, as well as many other cell types in the head, even in the absence of p53 function. Finally, to determine if the *fscn1aMO*-mediated cell death was associated with induction of known pro-apoptotic genes, we analyzed the expression of a subset of BH3-only pro-apoptotic genes and found specific induction of *puma* mRNA levels (S3 Fig). Puma is a strong inducer of apoptosis and can be activated in a p53-independent manner [34, 35]. Thus, despite previous studies demonstrating that *tp53* deficiency can alleviate nonspecific MO-induced apoptosis, these results show that MOs can also induce *tp53*-independent cell death, which may contribute to the widespread and severe NC phenotypes observed in *fscn1aMO* embryos.

## Discussion

### Morpholinos cause misleading NC migration and derivative formation phenotypes

Defects in craniofacial morphology have previously been shown to be an off-target phenotype caused by a variety of MOs [36]. We now show that NC cell behaviors and tissue morphogenesis are also particularly sensitive to MO-induced artifacts. Initial characterization of the *fscn1aMO* yielded promising results: 1) injection of the *fscn1aMO* significantly reduced endogenous Fscn1a protein levels, 2) wild-type *fscn1a* mRNA rescued the *fscn1aMO*-induced phenotypes in 5 dpf embryos, demonstrating specificity of the knockdown [12], 3) phenotypes were *p53*-independent and thus free from apoptosis-related off-target effects [11], and 4) *fscn1a* morphants had defects in filopodia formation which was expected based on the described molecular function of Fascin1 [32]. By all these criteria, the *fscn1aMO* appeared to be a specific knockdown reagent [12]. Indeed, the *fscn1a* morphant embryos also showed seemingly-specific defects in cranial collective NC migration and derivative formation that were consistent with earlier filopodia defects and gene expression changes (e.g., *dlx2*), but these phenotypes are not observed in the *fscn1a MZ* genetic mutant. Thus, in the absence of a null genetic mutant for comparison, *fscn1a* would have been mistakenly deemed critical for collective migration of all cranial NC streams and formation of all craniofacial elements.

### Morpholino-based studies contribute to inconsistencies in the NC field

The NC community has suffered a rash of inconsistencies in regard to gene function during NC development. To account for this, it has recently been suggested that mice either have genetic redundancies, insufficient gene targeting technologies (such as Wnt1-Cre) or differences in the temporal induction of NC genes [7]. Another possibility is the use of different techniques for genetic manipulation (e.g., gene knockdown in frog, chick and fish versus genetic mutant analysis in mice), but this was considered less likely as gene knockdown-induced NC phenotypes are typically rescued with wild-type mRNA [7]. Here we show that despite rescue with wild-type mRNA, and a host of other validating criteria, the *fscn1aMO* induced some wholly incorrect NC defects. Our study highlights the possibility that widespread use of antisense knockdown technologies, such as MOs in non-mammalian systems, has contributed to the inconsistencies in NC gene function observed between species. Our findings are also consistent with detailed examples of MOs that give seemingly specific developmental phenotypes, but ultimately do not recapitulate the corresponding genetic mutant (e.g., [18, 19, 37–39]). In addition, a recent study demonstrated that a large majority (~80%) of previously published MO-induced vasculature phenotypes are not observed in the corresponding genetic mutants [16]. While NC phenotypes were not analyzed in this study, it is interesting to note that some of the genes examined (*mmp2* and *ets1*) are examples for which MOs cause dramatic early NC phenotypes in non-mammalian systems [40, 41] while those observed in the corresponding mouse mutants are more selective [42, 43]. Future studies with zebrafish mutants will clarify the extent to which gene function is conserved in NC development, particularly in regard to genes for which only MO-based NC phenotypes are available.

It is important to note that a number of MOs faithfully phenocopy genetic mutants in zebrafish that are essential for early NC development, such as *sox10*, *tfap2a* and *foxd3* [44–47]. Indeed, in our opinion, the use of these morpholinos, particularly the *tfap2a/foxd3* MO combination that eliminates most NC cells [48, 49], should be considered equivalent to analyzing the corresponding double genetic mutant to determine a role for NC cells in development of other tissue types. In this case, the use of MOs can produce 100% of embryos lacking NC, compared to only 1/16 homozygous embryos generated from a genetic cross, thereby accelerating genetic interaction analysis, such as epistasis experiments. Nonetheless, even in these cases, establishing the single or double mutant phenotype first was essential for determining the fidelity of these MOs for subsequent studies.

### The power of mutant analysis to identify redundant pathways in NC development

A significant advantage of genetic mutant analysis is the ability to test the null hypothesis with confidence. That is, when no or low-penetrance NC phenotypes are observed in a null mutant, one can reliably conclude that the gene is either not required for a given developmental process, or acts redundantly with other genetic mechanisms. In contrast, one can never rule out the null hypothesis with MO-based knockdown experiments because small amounts of residual gene product may still be sufficient for normal function. Indeed, the absence of a MO-induced phenotype is often interpreted to be due to a non-functioning MO, promoting the practice of testing multiple MOs for a given gene until a phenotype is eventually observed, which generates an inherent bias toward inaccurate assessments of normal gene function.

The identification of NC-expressed genes that are partially or wholly dispensable for NC development is critical for the discovery of redundant genetic pathways. For example, mutation in either *foxd3* or *tfap2a* alone does not affect NC induction in zebrafish embryos but instead causes selective defects in NC migration and derivative formation [29, 45–47]. However, *foxd3*; *tfap2a* double mutants have essentially no *sox10*-positive NC cells or derivatives, showing that these two genes function in parallel pathways to control early NC induction [48, 49]. Similarly, the low penetrance and selective phenotypes of *fscn1a* mutants is ideal for identifying genetic enhancers that act together or in parallel to Fscn1a/filopodia to control directional NC migration, particularly in the mandibular NC stream. Such studies would not be possible with the *fscn1aMO* as it causes highly penetrant and severe defects in all cranial NC streams. Finally, a recent study also demonstrated that in at least two cases, compensatory/redundant genetic programs are activated in genetic mutants, but not the corresponding morphant [17]. While this study suggests some MOs could be used to identify compensatory mechanisms, in these cases genetic mutant analysis is still needed to determine the specificity and extent of genetic redundancy between two or more genes.

### Importance of clarifying conserved genetic mechanisms controlling NC development

Knowledge of the conserved genetic mechanisms controlling NC development is important for understanding the origin and evolution of many vertebrate-specific cell types and structures. For example, the identification and manipulation of genetic pathways controlling NC stem cell formation, migration and differentiation is a necessary first step in using NC stem cells for regenerative therapies, such as replacement of missing bone in congenital craniofacial syndromes. Moreover, NC mechanisms, such as the epithelial-to-mesenchymal transition (EMT), are re-activated to promote progression and therapeutic resistance in a number of human cancers (e.g., breast, lung, pancreas). A lucid and thorough understanding of the genetic mechanisms that govern NC development will be essential for translating basic discoveries in model organisms into treatments for human disease. Therefore, based on our findings, we suggest that clarifying the conserved genetic mechanisms controlling NC development will require comparison of single or double genetic mutant NC phenotypes between species, which is ultimately needed to guide identification of the most effective single or combination therapies to treat human NC disease and cancer.

## Supporting Information

S1 Fig Lateral views of 4 dpf wild-type (top panels) and *fscn1a MZ* (bottom panels) embryos injected with *coMO* or *fscn1aMO* as indicated. The *fscn1aMO* causes severe morphological defects in both wild-type and *fscn1a MZ* embryos, indicating that compensatory genetic pathways are not activated in *fscn1a* null mutants. All experiments in this figure were performed independently at least two times with similar results (n = 50 embryos/condition).
(TIF)

S2 Fig Cell death is minimal in *fscn1a MZ* cranial NC streams. Dorsal cranial views of 16 hpf *Tg(sox10*:*gfp); fscn1a MZ* embryos stained for GFP and activated Caspase-3. Boxed region in left panels is magnified in right panels. In all panels, anterior is to the left.
(TIF)

S3 Fig Upregulation of *puma* in *tp53* mutant embryos injected with *fscn1aMO*. Expression of *bad*, *bim*, *bid*, *puma* and *noxa* mRNA relative to *gapdh* in 24 hpf *tp53*^*M214K/M214K*^ embryos injected with *coMO* or *fscn1aMO*. Error bars represent the standard error of the mean (SEM) from three independent experiments, *p = 0.0057.
(TIFF)

S1 Table Quantitative real-time PCR primers and probes. (TIFF)
