# Supplementary figures and images for: Neural Crest Migration and Survival Are Susceptible to Morpholino-Induced Artifacts
Source: PLoS One. 2016 Dec 22;11(12):e0167278. doi: 10.1371/journal.pone.0167278 (PMC5179070; doi:10.1371/journal.pone.0167278)

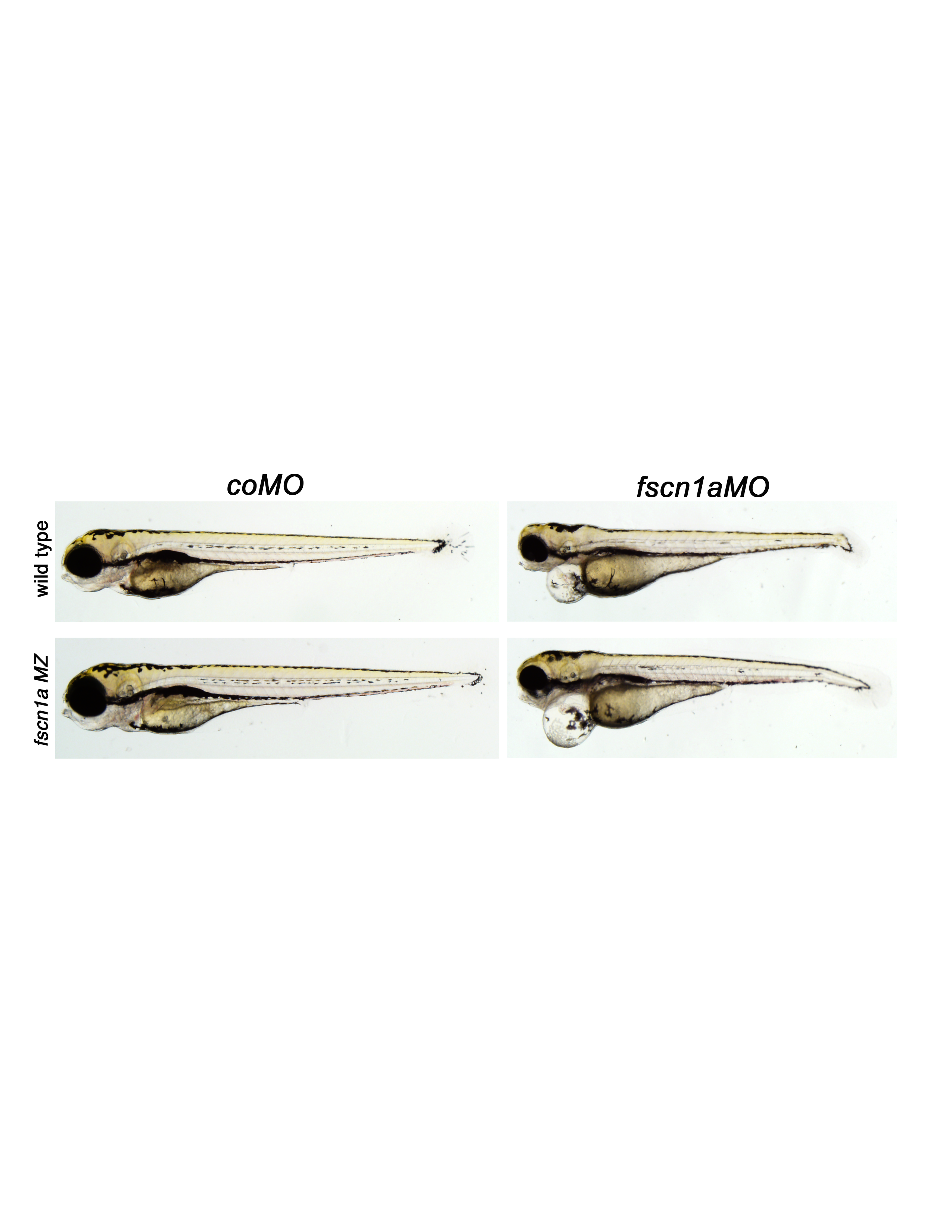

Supplement: S1 Fig — The fscn1aMO causes severe morphological defects in both wild-type and fscn1a MZ embryos, indicating that compensatory genetic pathways are not activated in fscn1a null mutants. All experiments in this figure were performed independently at least two times with similar results (n = 50 embryos/condition). (TIF) [file pone.0167278.s001.tif]

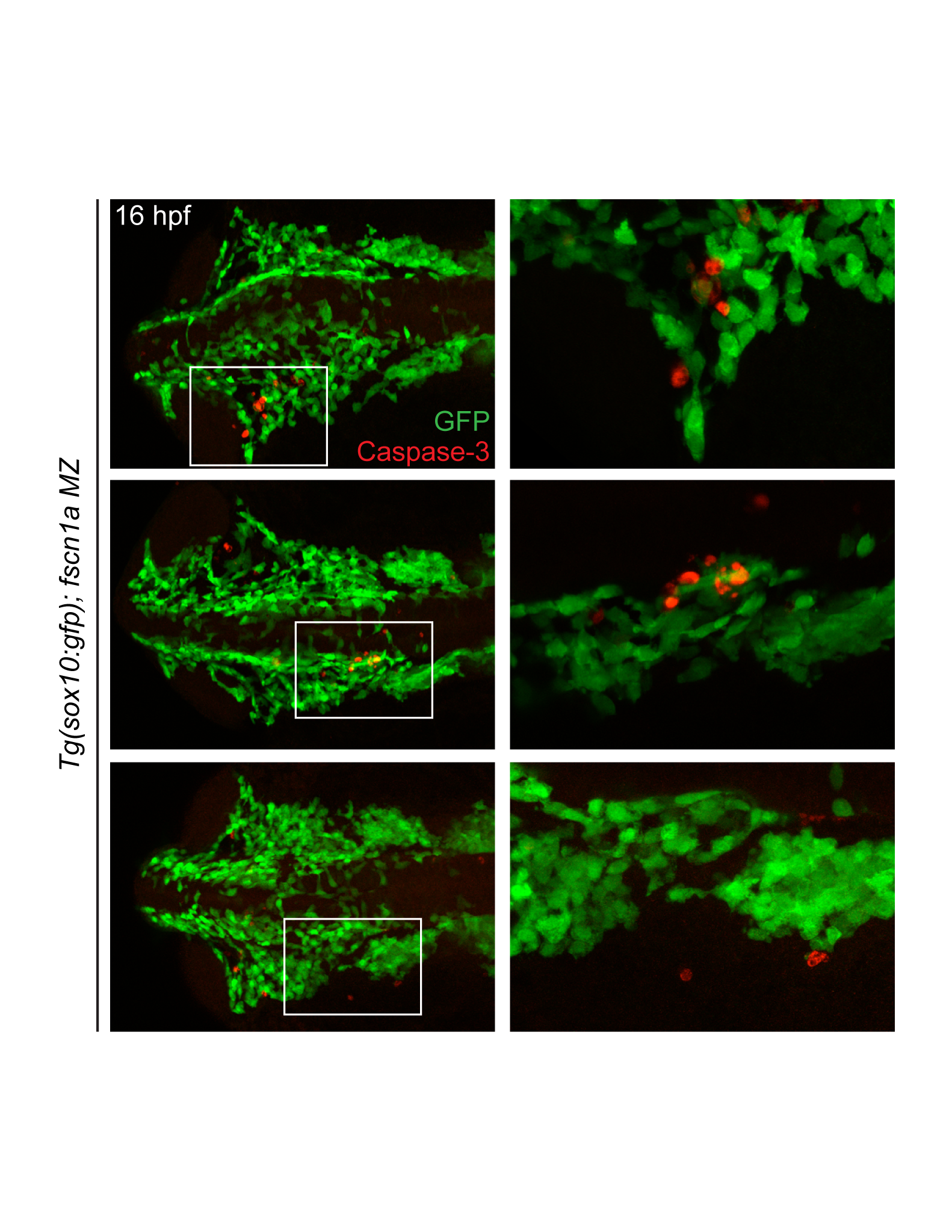

Supplement: S2 Fig — Dorsal cranial views of 16 hpf Tg(sox10:gfp); fscn1a MZ embryos stained for GFP and activated Caspase-3. Boxed region in left panels is magnified in right panels. In all panels, anterior is to the left. (TIF) [file pone.0167278.s002.tif]

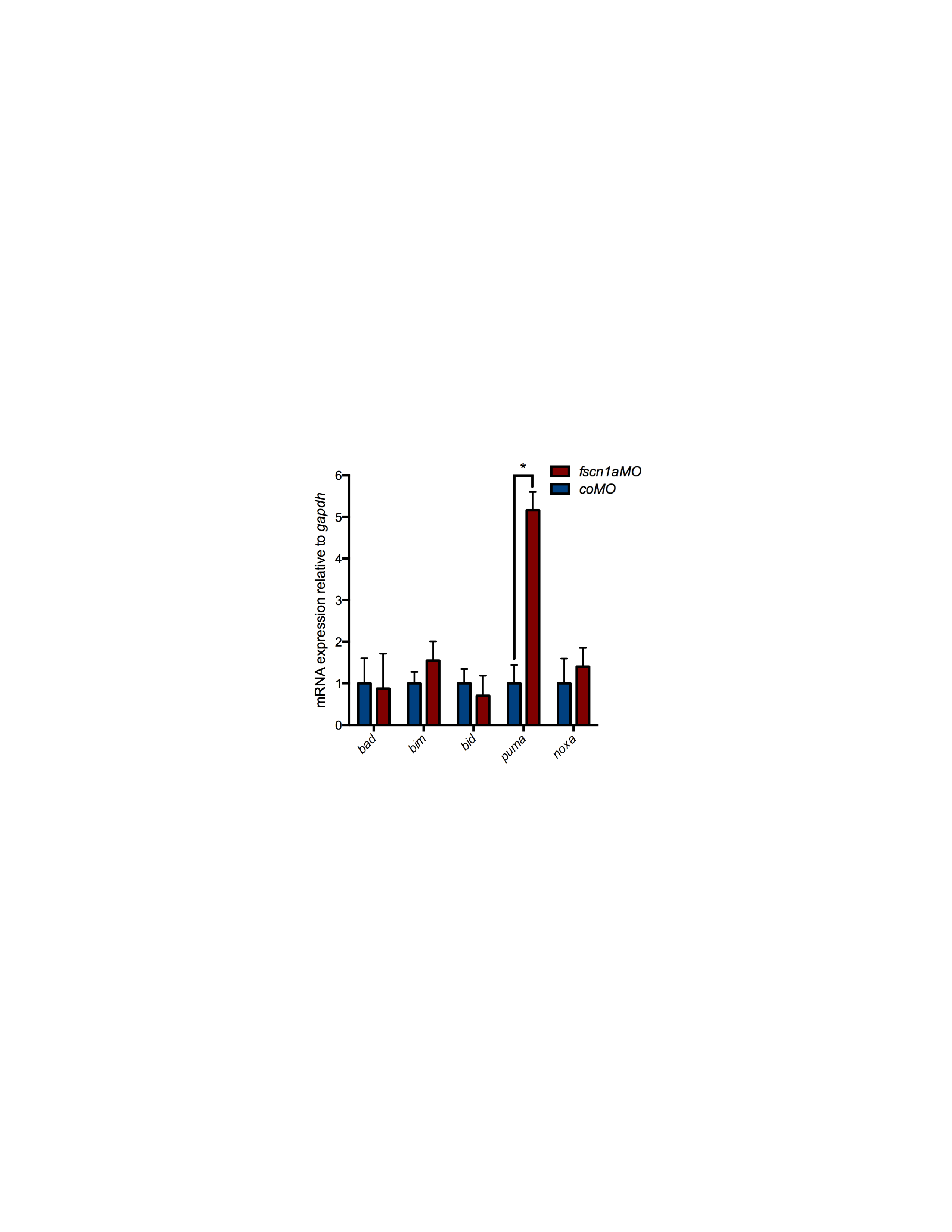

Supplement: S3 Fig — Expression of bad, bim, bid, puma and noxa mRNA relative to gapdh in 24 hpf tp53M214K/M214K embryos injected with coMO or fscn1aMO. Error bars represent the standard error of the mean (SEM) from three independent experiments, *p = 0.0057. (TIFF) [file pone.0167278.s003.tiff]

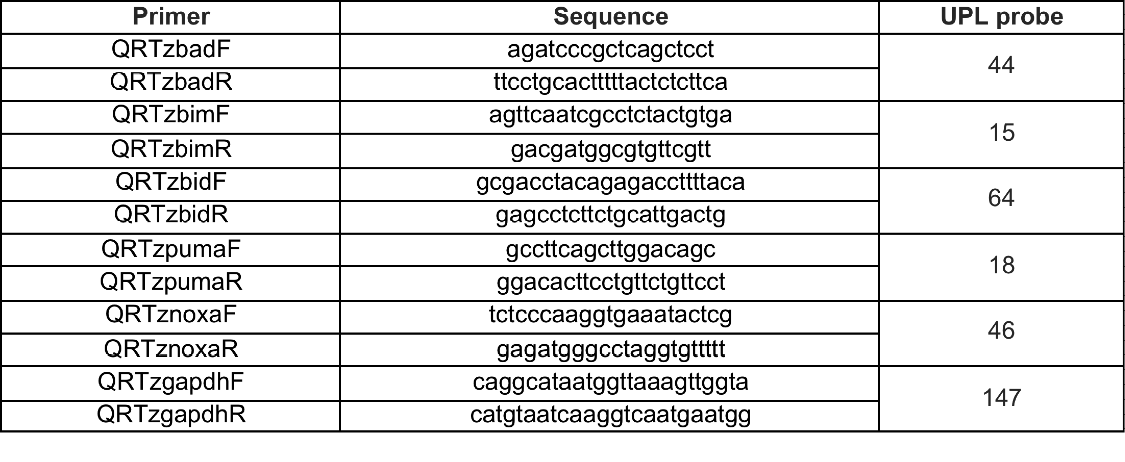

Supplement: S1 Table — (TIFF) [file pone.0167278.s004.tiff]
